# Fitting a Square Peg in a Round Hole: A Simple Case of Chest Pain

**DOI:** 10.5811/cpcem.2019.10.44141

**Published:** 2020-01-06

**Authors:** Mary E. McLean, Jennifer Beck-Esmay

**Affiliations:** * St. John’s Riverside Hospital, Department of Emergency Medicine, Yonkers, New York; † Mount Sinai St. Luke’s-Mt Sinai West, Department of Emergency Medicine, New York, New York

## Abstract

A 39-year-old female presents to the emergency department with chest pain and shortness of breath. Her electrocardiogram suggests ST-elevation myocardial infarction, but she has no atherosclerotic risk factors. She is gravida 4, para 4, and four weeks postpartum from uncomplicated vaginal delivery. She is diaphoretic and anxious, but otherwise her exam is unremarkable. Cardiac enzymes are markedly elevated and point-of-care echocardiogram shows inferolateral hypokinesis and ejection fraction of 50%. In this clinicopathological case, we explore a classically underappreciated cause of acute coronary syndrome in healthy young women.

## CASE PRESENTATION (Resident Presentation)

Paramedics place a call to the emergency department (ED). They are en route with lights and sirens! The patient is a 39-year-old female with 10/10 crushing left chest pain radiating to the left arm. Her field electrocardiogram (ECG) is suggestive of inferior ST-elevation myocardial infarction (STEMI). She receives full-dose aspirin and sublingual nitroglycerin in the ambulance with slight improvement in symptoms.

**Image 1:**
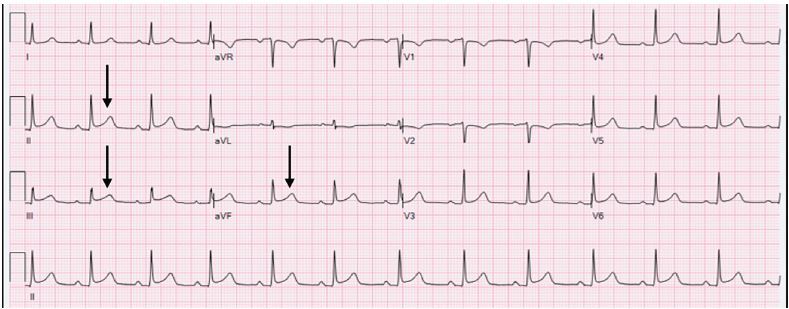
Initial emergency department electrocardiogram. Black arrows indicate ST elevations.

On arrival to the ED, the patient still has pain and associated diaphoresis, shortness of breath, cough, palpitations, headache, nausea, anxiety, and a feeling of impending doom. The ED ECG (Image 1) shows subtle ST elevations in II, III, and aVF, and a reciprocal T-wave inversion in aVL.

Peripheral intravenous access is obtained and the patient is placed on the cardiac monitor. Her systolic blood pressure is 128 millimeters of mercury (mmHg), diastolic blood pressure is 72 mmHg, heart rate is 88 beats per minute, respiratory rate is 20 breaths per minute, pulse oximetry is 98% on room air, and temperature is 36.8 degrees Celsius. Her body mass index is estimated at 25. She appears uncomfortable, diaphoretic, and anxious. There are no other abnormal cardiovascular, respiratory, abdominal, or neurologic findings on exam. There is no calf swelling, tenderness, or palpable cords. There are no sequelae of hyperlipidemia, diabetes mellitus, or other chronic illness. History is completed after critical stabilization actions.

The patient was resting at home when the symptoms began. She denies leg pain/swelling, hemoptysis, immobilization, surgery, exogenous hormone use, orthopnea, paroxysmal nocturnal dyspnea, syncope, or lightheadedness.

She receives regular medical care, and has never been diagnosed with any chronic illness or had surgery. She denies family history of coronary artery disease, sudden unexplained death, blood clotting disorders, or other chronic diagnosis. She has never used tobacco, alcohol, or drugs. She lives at home with her loving husband and four children, and denies psychiatric illness.

In fact, the only notable findings on history are regarding reproductive history. She is gravida 4, para 4 (G4P4), and four weeks postpartum. Pregnancy and vaginal delivery were uncomplicated and she has been breastfeeding without issue. She has help from multiple supportive family members, and she does not feel stressed.

Point-of-care echocardiogram reveals inferolateral hypokinesis and left ventricular ejection fraction (EF) of 50%, but no pericardial effusion, valvular abnormalities, or right heart strain. The chest radiograph (CXR) shows nothing acute. The total creatine kinase is 1344 units per liter and troponin I is 2.42 nanograms per milliliter, but remaining labs are normal (Table 1).

## CASE DISCUSSION (Attending Discussion)

I like to start thinking through any case with a little review. This patient was brought in by ambulance with chest pain, shortness of breath, and diaphoresis. Her vitals were stable and she received nitroglycerin and aspirin, but ECG showed persistent inferior ST elevation and reciprocal changes. At this point, the case seems quite simple: the STEMI code should have been activated, and the patient should have had immediate coronary angiography. But that’s not what happened for this patient. Instead, she had a CXR, echocardiogram, and lab work prior to diagnosis. Why didn’t she get a speedy door-to-balloon time? Maybe because the patient is a woman? Studies have shown that women are less likely than men to undergo coronary angiography or revascularization and, specifically, they are less likely to receive timely revascularization.^1^

Implicit gender bias may have played a role in this case and there are probably some very good reasons why. So let’s step back and review the most significant details. This patient was a previously healthy 39-year-old female, whose chest pain presentation was complicated by the fact that she was G4P4 and just four weeks postpartum. Usually when a postpartum woman presents with chest pain or shortness of breath, there are two obvious diagnoses to consider: pulmonary embolism (PE) and postpartum cardiomyopathy.

PE is a leading cause of pregnancy-related death in the developed world.^2^ The incidence of venous thromboembolism (VTE) is about 13 in 10,000 pregnancies, with half occurring before delivery and half in the postpartum period. The increased risk of VTE continues for 6–12 weeks postpartum. Whether a patient is recently pregnant or not, this diagnosis is always on my differential for chest pain and/or shortness of breath in a patient with lungs that are clear to auscultation. However, this patient has no clinical signs or symptoms of deep venous thrombosis, and she has normal vital signs. Healthy young patients may have good cardiovascular reserve and may not show any abnormalities until later in the course of their illness, so I would keep PE on the differential for now.

No thought exercise on pregnancy and chest pain would be complete without mentioning amniotic fluid embolism. This is similar to a pulmonary VTE, but it is due to amniotic fluid entering the maternal pulmonary circulation. This presents with a classic triad of hypoxia, hypotension, and coagulopathy. Most of these occur during labor; but about one third happen during the immediate postpartum period.^3^ This patient has no hypoxia, hypotension, or coagulopathy and is outside the expected time frame for amniotic fluid embolism, meaning we can likely remove it from the differential.

Next, let’s think about peripartum cardiomyopathy. This typically occurs in the postpartum period and is marked by left ventricular dysfunction and heart failure. Research has suggested that peripartum cardiomyopathy is caused by vascular dysfunction, triggered by late-gestational maternal hormones.^4^ According to the definition from the European Society of Cardiology, these patients have a reduced EF, usually less than 45%, toward the end of pregnancy or in the months after delivery. Clinically, this patient does not look like she has heart failure. On exam she has clear lungs and no peripheral edema, and on CXR there is no pulmonary edema; so this makes heart failure less likely.

We have another clue in the case that can help us think about both PE and cardiomyopathy: the echocardiogram. There was no right heart strain, and a 50% EF. It seems unlikely to have a PE large enough to cause an elevated troponin without signs of right heart strain, and the 50% EF isn’t quite low enough for cardiomyopathy. The combination of her clinical picture and the echocardiogram allows us to remove PE and cardiomyopathy from our differential.

Because neither of these likely diagnoses fit in this patient, we turn again to the ECG, which shows a STEMI. On top of that, the inferolateral hypokinesis on her echocardiogram is a wall motion abnormality that fits the ST-elevation distribution on her ECG. This got me thinking: why would a healthy young postpartum woman have a STEMI? Pregnancy-related acute myocardial infarction (MI) is super rare! It complicates only 6.2 of 100,000 pregnancies.^5^ Wait, is there a differential diagnosis for STEMI?

The *Fourth Universal Definition of Myocardial Infarction* Task Force provided an international consensus on the classification of myocardial injury and infarction in 2018.^6^ They defined five types of MI.

MI types 4 and 5 are those associated with revascularization procedures, either percutaneous coronary intervention (PCI) or coronary artery bypass grafting (CABG), respectively. This patient had no recent procedure, so she couldn’t have a type 4 or 5 MI.

Type 3 MI occurs when a patient suffers cardiac death, with symptoms or ECG changes that suggest MI, but who die before we can obtain biomarkers. This scenario clearly doesn’t fit our alive patient.

Type 2 MI is due to an imbalance between myocardial oxygen supply and demand, unrelated to coronary artery disease. This is what we think of clinically as “demand ischemia.” Supply and demand imbalance causes MI in a variety of ways, and some of these are worth exploring (Table 2).

This patient does not have hypertension, respiratory failure or shock; so we can immediately remove those from our differential. Her ECG shows normal sinus rhythm, so we can rule out the tachy- or brady-dysrhythmias. According to her blood work, she is not anemic. Her CXR shows a normal mediastinum, and while that does not entirely rule out aortic dissection, it certainly makes it less likely. That leaves coronary vasospasm (Prinzmetal’s angina) and coronary artery dissection (CAD).

Coronary spasm was first reported by Prinzmetal et al. in the 1950s when they demonstrated reversible myocardial ischemia accompanied by ST-segment elevation on the ECG. Coronary artery spasm is defined as “dynamic, transient reduction in the luminal diameter of the epicardial coronary arteries due to increased vasomotor tone leading to myocardial ischemia.” It causes only 1.8% of pregnancy-related MIs,^7^ which is pretty rare but not impossible in this patient.

Next, let’s turn to CAD. Spontaneous coronary artery dissection (SCAD) is a non-traumatic, non-iatrogenic epicardial CAD. While the cause of pregnancy-associated SCAD is not fully understood, we think the hormonal changes of pregnancy may compromise the arterial wall architecture. Importantly, SCAD isn’t limited to the peripartum woman; it’s a major cause of MI overall in women ≤50 years of age.^8^

The last type of MI (type I) is caused by plaque rupture or erosion leading to thrombus formation in a patient with atherosclerotic coronary artery disease. Our patient has no known atherosclerotic risk factors, making type 1 MI less likely.

We now have three causes of STEMI that remain on this patient’s differential: coronary artery vasospasm, CAD, and classic plaque rupture with thrombus formation. The diagnostic test in each case is the same: the patient needs coronary angiography. To diagnose this patient without that study, as asked to do for a clinicopathological case, then becomes about numbers and odds. Pregnancy-associated SCAD is the most common cause of MI among patients who are pregnant or postpartum, accounting for 43% of acute MI in the peripartum population,^9^ making this the most likely cause of her MI.

### Clinical Diagnosis

Acute ST-elevation myocardial infarction due to pregnancy-associated spontaneous coronary artery dissection.

## CASE OUTCOME (Resident Presentation)

Given this patient’s STEMI in the setting of multiparity and recent postpartum status, a provisional diagnosis of SCAD was made by the ED provider. The patient was further managed in the ED with a heparin bolus and drip, and metoprolol. Cardiology was called for immediate consultation. Over the following four hours, the ST elevations improved on repeat ECG and the troponin climbed slightly but thereafter plateaued. The patient was admitted to the cardiac care unit (CCU) and was prepared for cardiac catheterization.

**Image 2:**
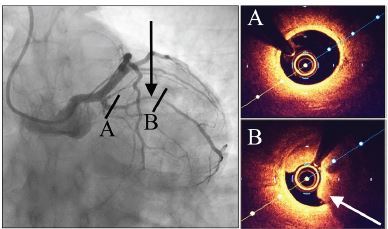
Coronary catheterization images. Fluoroscopy is shown on the left, and intracoronary optical coherence tomography (OCT) is shown on the right. The black arrow indicates the lesion on angiography. The white arrow indicates intramural hematoma on OCT. Letters A and B signify locations along the lesion.

Coronary angiography (Image 2) revealed a long but subtle lesion in a large branch of the right coronary artery. To better characterize this lesion, the adjunctive intravascular ultrasound imaging modality optical coherence tomography (OCT) was used during cardiac catheterization. The OCT of the lesion demonstrated a large intramural hematoma with intimal disruption. There was 75% obstruction of the true lumen at the time of catheterization.

The patient stayed in the CCU for several days on the heparin drip and metoprolol. Her symptoms resolved, and her lab values and ST elevations normalized. Her ECG on discharge from the hospital showed a persistent T-wave inversion in aVL. She continued daily metoprolol and baby aspirin, and declined the option of clopidogrel because she was breastfeeding. She had a negative outpatient workup for fibromuscular dysplasia, and has had no recurrence of symptoms.

## RESIDENT DISCUSSION

### Pathophysiology

SCAD is defined as “separation of the coronary arterial wall by intramural hemorrhage creating a false lumen, with or without an intimal tear.”^10^ It develops in much the same way as aortic dissection; an intimal tear often results in high-pressure accumulation of hematoma between the vessel layers. Alternatively, the vasa vasorum can bleed between weakened vessel layers to create intramural hematoma. If this hematoma compresses the true lumen, it can cause complete or near-complete coronary vessel occlusion, resulting in STEMI or non-STEMI, respectively. There is an atherosclerotic variant of SCAD that tends to be self-limited by medial scarring and atrophy, but non-atherosclerotic SCAD is the real killer; it results in more extensive and severe dissection and has an acute mortality rate of 28–82%.^11^,^12^

In 2018, *Circulation* published an American Heart Association (AHA) scientific statement noting that SCAD is more common than previously believed. Typical SCAD patients are healthy young women without conventional atherosclerotic risk factors. The AHA also expressed that SCAD must be evaluated and treated differently from atherosclerotic acute coronary syndrome (ACS).^8^ These statements drive home three important points:

- Epidemiology: SCAD is more common than previously thought
- Demographics: SCAD and type 1 MI patients differ markedly in age and risk factors.
- Management: SCAD and type 1 MI clinical management differs markedly.

### Epidemiology

SCAD is more common than previously thought. According to recent literature, it accounts for up to 4% of ACS overall, and for up to 35% of ACS occurring in women aged 50 and younger.^8^ A retrospective review of an 32,869-patient angiography database found that women constituted 77% of SCAD cases, and all of these women had undergone at least one prior pregnancy.^13^ Historical underdiagnosis of SCAD is likely due to the subtle coronary angiography findings of long and diffusely narrowed coronary vessel segments. This triggered the need for OCT, which came into use in the mid-1990s. The true incidence and prevalence of SCAD remain unknown.^14^

### Demographics

Although patients with SCAD present with the same symptoms as those with atherosclerotic ACS, the demographics differ markedly. In the ED, this means we have a different population of chest pain patients to be worried about: healthy young women.

SCAD is associated with fibromuscular dysplasia and other predisposing arteriopathies. Aside from genetic and structural abnormalities, the mechanism for vessel wall weakening is poorly understood. A widely accepted theory is that chronically elevated circulating hormone levels increase the risk of SCAD in multiparous (≥4 births), pregnant, and/or postpartum women. Interestingly, postpartum status was the sole risk factor in 18% of women, with a mean postpartum period of 38 days at the time of SCAD diagnosis.^15^

### Management

Although SCAD is managed differently from atherosclerotic ACS, the initial ED workup is the same. The ECG for these patients often shows STEMI (25–50% of cases) or non-STEMI. The left anterior descending coronary artery is most commonly involved, and multivessel disease is not rare.^13^ Cardiac enzymes may be elevated, echocardiogram can reveal left ventricular dysfunction, and up to 14% of cases are complicated by ventricular dysrhythmias.^16^

Medical management is strongly preferred for SCAD. While in the ED, SCAD patients should still get aspirin, nitroglycerin, and heparin whenever applicable. They *should not* routinely get thrombolytics; there are prior descriptions of thrombolysis-precipitated extension of the dissection or even rupture leading to cardiac tamponade.^17^ SCAD patients should undergo coronary angiography with OCT for definitive diagnosis. Stenting is reserved for severe cases. Balloon angioplasty can rupture the compromised vessel wall or cause hematoma propagation. A Mayo Clinic case series of 189 patients showed technical failure in 53% of patients initially managed with percutaneous coronary intervention (PCI).^18^ CABG may be reasonable for persistent STEMI, severe symptoms, or cardiogenic shock.

Beyond the acute phase, SCAD patients are typically worked up for fibromuscular dysplasia, connective tissue disorders, and systemic inflammatory conditions. Long-term management includes a beta blocker, statin, and daily baby aspirin and/or clopidogrel. As for long-term prognosis, the 10-year rate of major adverse cardiac events (death, heart failure, MI, and SCAD recurrence) was found to be 47%.^16^

## FINAL DIAGNOSIS

Pregnancy-related spontaneous coronary artery dissection.

## KEY TEACHING POINTS

- SCAD is historically underdiagnosed and accounts for up to 35% of MI in women aged ≤50 years, especially multiparous and peripartum women
- Medical management is strongly preferred (aspirin, heparin, and metoprolol), but do not thrombolyse these patients
- SCAD patients need coronary angiography for definitive diagnosis; but reserve stents and CABG for unstable patients or persistent STEMI, and do not perform balloon angioplasty
- Treat cardiogenic shock the same as all other cardiogenic shock (PCI, CABG, intra-aortic balloon pump, extracorporeal membrane oxygenation, left ventricular assist device, and implantable cardioverter-defibrillator)

## Figures and Tables

**Table 1:** Emergency department laboratory results. Abnormal values are flagged with (H).

Test	Value	Reference
Hematology (serum)		
White blood cell count	9.9 K/mm^3^	4.0–10.0 K/mm^3^
Red blood cell count	4.78 million/uL	3.6–5.2 million/uL
Hemoglobin	13.8 g/dL	10.7–15.3 g/dL
Hematocrit	41.9%	32.4–45.2%
Mean cell volume	87.7 fL/cell	80–96 fL/cell
Mean corpuscular hemoglobin	28.9 pg	25.7–33.7 pg
Mean corpuscular hemoglobin concentration	33 g/dL	32.0–36.0 g/dL
Red blood cell distribution width	13.6%	11.6–15.6%
Platelet volume	238 K/mL	134–434 K/mL
Mean platelet volume	10 fL	7.5–11.1 fL
Chemistry (serum)		
Sodium	139 mmoles/L	136–145 mmoles/L
Potassium	3.9 mmoles/L	3.5–5.1 mmoles/L
Chloride	103 mmoles/L	98–107 mmoles/L
Carbon dioxide	22 mmoles/L	21–32 mmoles/L
Anion gap	14 mmoles/L	8–16 mmoles/L
Blood urea nitrogen	12 mg/dL	7–18 mg/dL
Creatinine	0.7 mg/dL	0.55–1.3 mg/dL
Glucose	106 mg/dL	74–106 mg/dL
Calcium	8.5 mg/dL	8.5–10.1 mg/dL
Total bilirubin	0.4 mg/dL	0.2–1.0 mg/dL
Aspartate aminotransferase	35 U/L	15–37 U/L
Alanine transaminase	39 U/L	13–61 U/L
Alkaline phosphatase	83 U/L	45–117 U/L
Total protein	6.9 g/dL	6.4–8.2 g/L
Albumin	3.7 g/dL	3.4–5.0 g/L
Total creatine kinase	1,344 U/L (H)	30–170 U/L
Troponin	2.42 ng/mL (H)	0.00–0.05 ng/mL
Thyroid function		
Free thyroxine	1.1 ng/dL	0.9–2.4 ng/dL
Thyroid stimulating hormone	2.24 mIU/L	0.83–1.09 mIU/L
Coagulation		
Prothrombin time	12.1 s	9.7–13.0 s
International normalized ratio	1.07	0.83–1.09
Urinalysis		
Color	Yellow	None
Appearance	Clear	None
Potential hydrogen	6	5.0–8.0
Specific gravity	1.019	1.010–1.035
Protein	Negative	Negative
Glucose	Negative	Negative
Ketones	Negative	Negative
Test	Value	Reference
Blood	Negative	Negative
Nitrite	Negative	Negative
Bilirubin	Negative	Negative
Urobilinogen	Negative	0.2–1.0 mg/dL
Leukocyte esterase	Negative	Negative

*K*, thousand; *mm^3^*, cubic millimeter; *uL*, microliter; *g*, gram; *dL*, deciliter; *fL*, femtoliters; *pg*, picograms; *mmoles*, millimoles; *L*, liter; *mg*, milligram; *U*, units; *ng*, nanogram; *mL*, milliliter; *mIU*, milli-international unit; *s*, second.

**Table 2:** Causes of type 2 myocardial infarction.

Anemia
Aortic Dissection
Aortic Valve Dissection
Arrhythmias
Coronary Artery Dissection
Coronary Vasospasm
Hypertension
Left Ventricular Hypertrophy
Respiratory Failure
Shock
